# Genomic Instabilities, Cellular Senescence, and Aging: *In Vitro, In Vivo* and Aging-Like Human Syndromes

**DOI:** 10.3389/fmed.2018.00104

**Published:** 2018-04-17

**Authors:** Gabriel Lidzbarsky, Danielle Gutman, Huda Adwan Shekhidem, Lital Sharvit, Gil Atzmon

**Affiliations:** Department of Human Biology, University of Haifa, Haifa, Israel

**Keywords:** aging, cellular senescence, DNA damage, telomeres, epigenetics

## Abstract

As average life span and elderly people prevalence in the western world population is gradually increasing, the incidence of age-related diseases such as cancer, heart diseases, diabetes, and dementia is increasing, bearing social and economic consequences worldwide. Understanding the molecular basis of aging-related processes can help extend the organism’s health span, i.e., the life period in which the organism is free of chronic diseases or decrease in basic body functions. During the last few decades, immense progress was made in the understanding of major components of aging and healthy aging biology, including genomic instability, telomere attrition, epigenetic changes, proteostasis, nutrient sensing, mitochondrial dysfunction, cellular senescence, stem cell exhaustion, and intracellular communications. This progress has been made by three spear-headed strategies: *in vitro* (cell and tissue culture from various sources), *in vivo* (includes diverse model and non-model organisms), both can be manipulated and translated to human biology, and the study of aging-like human syndromes and human populations. Herein, we will focus on current repository of genomic “senescence” stage of aging, which includes health decline, structural changes of the genome, faulty DNA damage response and DNA damage, telomere shortening, and epigenetic alterations. Although aging is a complex process, many of the “hallmarks” of aging are directly related to DNA structure and function. This review will illustrate the variety of these studies, done in *in vitro, in vivo* and human levels, and highlight the unique potential and contribution of each research level and eventually the link between them.

## General Introduction

During an organism’s lifetime, cells are constantly exposed to exogenous and endogenous stressful agents. Cells can cope with these stressors by various response mechanisms, or in case of irreversible damage, programmed cell death (apoptosis), or permanent cell-cycle arrest (cellular senescence). Cellular senescence is characterized by a halt in cellular replication, accompanied by a specific molecular phenotype (1–3). This phenotype can be the result of a few factors, such as accumulation of DNA damage, telomere attrition, and various epigenetic alterations (4).

In this review, we will highlight the major efforts to unveil the role of senescence in healthy aging by three main strategies: *in vitro, in vivo*, and human. Each strategy has advantages and limitations, yet when stratified and combined can elucidate molecular and physiological mechanisms and phenotypes, in general, and in healthy aging in particular.

## Cellular Senescence and Physiological Aging

**Figure 1:**
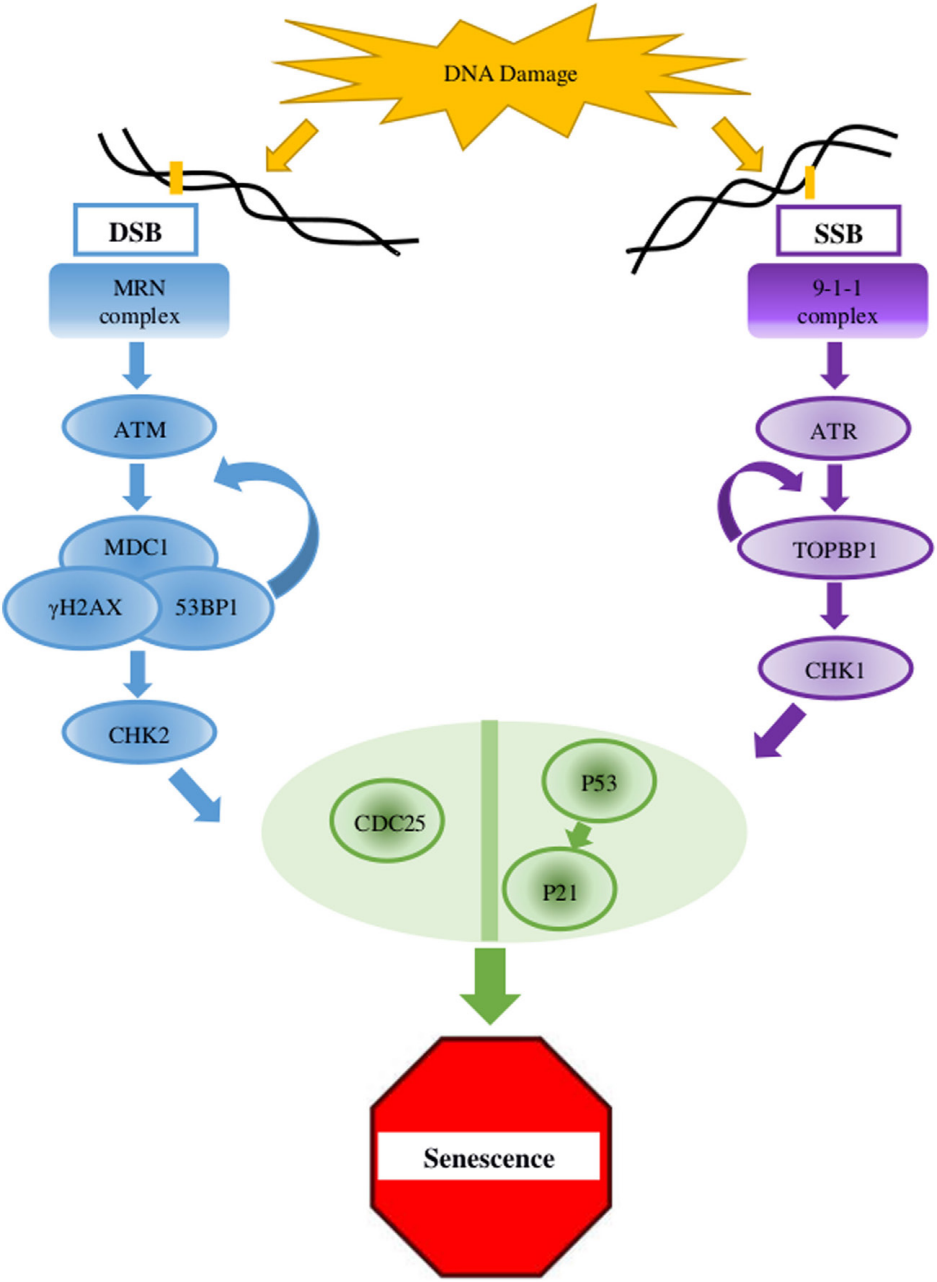
Key elements in the DNA damage response (DDR) pathway. In case of double-strand breaks (DSB), the DNA damage sensor MRN complex recruits the protein kinase ATM which activates γH2AX at the damaged site. γH2AX connects to MDC1, and this complex amplifies the activity of the MRN complex which, in a positive feedback, amplifies the ATM activity and the dispersal of γH2AX along the chromosome. MDC1 and 53BP1 further mediates the activation of CHK2 which carries the signal to distant locations on the genome. For single-strand breaks (SSB), the protein kinase ATR is activated and amplified by the 9-1-1 complex and TOPBP1, which also mediates the activation of CHK1. The signaling pathway cascades toward the key factors p53 and CDC25. When the lesion is repaired, the DDR complexes are dismantled (2, 4, 9).

The aging process is a complex trait that combines different biologic levels. Aging at the organism level includes failure to maintain internal environment and regular function, alongside increased susceptibility to diseases. Aging at the tissue level may involve, for example, chronic inflammation, which in turn contributes to cardiovascular and neurodegenerative disorders (5). The mechanisms of aging are affected by cellular and non-cellular pathways. The buildup of chronic stress, for example, is significant for the aging phenotype, but it is an organism-level phenotype (6). Structural deterioration of the body will influence an organism’s ability to forage, resulting in bad nutritional state that in turn will speed the aging process. Cellular senescence is one of the cellular pathways contributing to organismal aging. This process is triggered by several factors such as accumulation of DNA damage, telomere attrition, and various epigenetic alterations and involves the activation of permanent cell-cycle arrest. Yet, unlike quiescence and other kinds of no-proliferation conditions, it is followed by a typical gene expression, metabolic activity, and a senescence-associated secretory phenotype (SASP). Cellular senescence is a multistage path. Once activated, the arrested cells shift from unstable to steady cell-cycle arrest, in a procedure that involves p21, p16^Ink4a^, and p53 (Figure 1). Next, alterations in chromatin methylation are generated. Senescent cells can accumulate in tissues and organs and can ultimately result in tissue lesions that will cause organ dysfunction (7, 8), and thus the cellular processes can lead to organism-level decay in function and health.

## From Cell Culture to Human Subjects: Strategies in Aging Research

### *In Vitro* 

Cell cultures are used in biological research since 1912. Carrel (10) isolated and cultured chicken cells to study aging processes (10). He concluded that the single cell is immortal, and aging and death are multicellular organism-related phenotypes. It was not until 1961 that Hayflick and Moorehead proved that Carrel was wrong and normal cells have limited proliferation capability in culture (10–12), also known as the Hayflick limit. Hayflick and Moorehead also discovered that normal cells looked “old” after they exhausted their replication potential. They speculated that single-celled replicative senescence contributed to the organism’s aging (11), which promoted the use of cell cultures to study aging processes in the full organism (12). Since the study by Hayflick and Moorehead, *in vitro* studies became the basis for every study in human biology. *In vitro* studies enable comparisons between many types of cells including mesenchymal stem cells, peripheral blood mononuclear cells, lymphoblast cells, muscle satellite cells (SCs), skin fibroblasts, endothelial cells, and embryonic stem cells, cultures from different organisms and different donor’s ages, enabling use for studying the genetics and biology of aging. Another advantage of *in vitro* studies is the capability to easily perform manipulations and treatments directly on the cells and to study the responses isolated from the original environment. The biggest limitation of *in vitro* studies is the translation to a whole organism (13). In culture, cells “behave” differently due to the loss of the cross talk between cells and the extracellular matrix from other regions in the body (such as immune system or hormonal signals). Though it helps with eliminating background pathway signaling noise when investigating certain mechanisms or pathways, it is a setback when trying to translate the effect of a manipulation or treatment to the whole organism. In attempt to compensate for the main *in vitro* limitation (i.e., translation drawback), researchers turn to *in vivo* (animal model) studies.

### *In Vivo* 

*In vivo* studies can further test the effect of a manipulation or treatment, either targeted or scattered, on the whole organism. Most of these biological models offer many advantages over humans, for instance, their basic biology and genomes are well documented and are easier to manipulate genetically. Furthermore, they have much shorter life spans than humans, enabling longitudinal studies, while ethical issues, long natural life span, environmental influences, genetic heterogeneity, and various other limiting factors complicate the use of human subjects in aging research. Regardless of the advantages listed earlier and the eminent contribution to our understanding of the aging process, the use of animal models in aging studies has its own limitations. Aging is not a simple process, and there is no genuine agreement about what it is and how to define it (14, 15), despite the agreement on being a multifactorial and complex phenomenon. Additionally, there is conflicting evidence about aging as a process that is similar across all organisms or particular to each species (15, 16). Therefore, it is important to draw attention to the fact that animal models are usually chosen for convenience rather than for specific features applicable to human aging. Hence, choosing the suitable animal model to answer the specific question we aim to understand is of high importance in these types of studies. Among the most prevalent aging model organisms are *Saccharomyces cerevisiae, Caenorhabditis elegans, Drosophila melanogaster*, and *Mus musculus*. As a single-celled organism, *S. cerevisiae* is easily grown, manipulated, and observed; together with a well-characterized genome that bares much resemblance to bigger and more complex organisms, this model organism among others is a convenient platform for the study of the aging phenotype. Another important model system for studying a range of biological processes, including aging, is the nematode *C. elegans*. *C. elegans* has a short adult life span of ~2 weeks and a well-documented anatomy which is visible using a microscope. This enables easy observations of aging-related changes in the whole organism, in specific tissues and organs, and even on molecular and cellular levels (17–21). The classic genetic model organism, *D. melanogaster*, is also used in the study of aging. Studies conducted in these flies have identified single gene mutations that influence their life span. One of the strengths of Drosophila as a model organism is the capability to illustrate how genes that have an established role in regulating organismal life span particularly influence cellular and tissue function, how they work together, and how their tissue-specific functions might be linked (22–25). That said, Drosophila is far from being a good model for human aging as they share only 60% of the human genome. A better similarity is achieved with *M. musculus*, the mouse. It is the most commonly used model in biological research for various reasons. Mice are small, have a short generation time, and an accelerated life span which means they are not expensive and require only little space and time, compared to larger animal models. Another important reason is the fact that the mouse genome is well documented and can be easily manipulated. In addition, they are biologically similar to humans, exhibiting many of the same diseases and conditions. Nevertheless, mice do not develop several important age-related diseases naturally (e.g., atherosclerosis and diabetes), a fact that limits their potential as an aging model. All the organisms described earlier are short-lived, which is one of their desired traits as model organisms. However, that may not be appropriate for the study of human aging. Thus, in recent years there have been more studies conducted on non-model long-living organisms such as the naked mole rats and bats, which may be more appropriate models in understanding healthy human aging. The naked mole rat (*Heterocephalus glaber*) is a very important non-model organism in cancer and aging studies. This subterranean, mouse-sized, eusocial rodent is known as the longest-living rodent, living 4–17 years in the wild and with captive individuals demonstrating exceptional longevity that exceeds 30 years (26)—almost an order of magnitude longer than mice. Moreover, until a few years ago no cancer cases were reported in NMRs, and researchers failed to induce tumorigenesis, placing this rodent as a novel model for cancer studies. Bats are the second most speciose mammalian order after rodents. Little brown bats (Myotis) are the smallest bats (3–30 g) with the highest longevity records (*Myotis myotis* live for 37.1 years and *M. brandti* live for 41 years). Nevertheless, longevity is generally high in all bat lineages, which makes them an interesting model in biogerontology. One of the most interesting non-model organisms adopted for aging research is the Bowhead whale (*Balaena mysticetus*), which is estimated to be the longest-living mammal, reaching the age of ~200 years and also one of the biggest species, with length and weight of 20 m and 100 tons (6, 27). Bowhead whales live in arctic environment and are well adapted to these harsh surroundings. They are considered to be resistance to cancer and age-related diseases, and thus, though research is very technically complicated, the study of Bowhead whale in the context of longevity could improve our understanding of molecular mechanisms of healthy aging (27).

### Human Aging-Like Syndromes

The limitations of *in vitro* and *in vivo* studies, and the great power of inferring from human studies on the human population, lead researchers to focus on aging-like human models. There are obvious moral and ethical limitations when working with human subjects, for this reason, most information on human aging was obtained from various progeroid syndromes, especially Hutchinson–Gilford progeria syndrome (HGPS) and Werner syndrome (28). These genetic conditions offer a glimpse into the molecular and physiological mechanisms of the aging cell and body, yet they do not capture the entire complexity of the aging and senescence phenotypes. Another approach for this purpose is using genome- and epigenome-wide association studies (GWAS and EWAS, respectively), which utilize the great improvement in whole genome sequencing technologies. Such studies have highlighted aging-related genes such as *APOE* (apolipoprotein E) (29–31) and have alleviated the dependency on *in vitro* and *in vivo* models by using direct human samples.

## Age-Related DNA Damage and DNA Damage Response (DDR) Activity

Age-related accumulation of DNA damage has been studied thoroughly, showing correlation between age and damage levels or mutation frequency (32, 33). In the presence of DNA lesions or abnormalities, the DDR, a complex multigenic pathway, is activated and can eventually lead to cell cycle arrest (Figure 1) (2, 4, 9). In older organisms, accumulation of DNA damage and loss of regenerative potential consequently increase the number of senescent cells, leading to aging cells, tissues, organs (4), and inevitable death (2, 34, 35). The general term DNA damage encompasses different types of lesions in the DNA, including large chromosomal lesions such as double-strand breaks (DSBs) and small, local lesions such as single-strand breaks (SSBs) and mismatched bases. To prevent the deleterious effect of these lesions, cells have evolved four DNA damage repair mechanisms. For large DSBs, such as the case in DSBs, cells utilize homologous recombination (HR) or non-homologous end joining (NHEJ). SSBs are resolved *via* the base- or nucleotide-excision repair pathways (BER and NER, respectively) (33, 36), and mismatched bases are corrected by the mismatch repair (MMR) mechanism (37).

### BER Reactive Oxygen Species (ROS)-Related DNA Damage Repair Efficiency, *In Vitro*

Wang et al. (38) tested lens samples isolated from age-related cataract (ARC) patients and age-matched patients with unrelated eye diseases (38). ARC was found to be affected by ROS and oxidative DNA damage, which is repaired by the BER pathway. The study showed that in ARC patients the expression levels of 8-oxoguanine DNA glycosylase (OGG1), a core member of the BER pathway, were significantly low. In addition, hypermethylation was demonstrated in the first exon of *OGG1*, hinting at the role of faulty DDR in the formation of ARC. Age-related BER activity was also studied with human foreskin fibroblasts derived from 20 to 64-year-old healthy donors, with similar results showing BER efficiency decrease with age. However, among several BER-related factors that were assessed, only Polβ (DNA polymerase beta) and XRCC1 (X-Ray Repair Cross Complementing 1) showed correlation between expression levels and age. In addition, a negative correlation was observed between age and the expression of Sirtuin 6 (SIRT6), which is connected to DNA maintenance and DSB repair (39), demonstrating a correlation between SIRT6 expression levels and BER quality. While overexpression of SIRT6 increased BER activity, SIRT6 knockout decreased BER activity, in the human foreskin fibroblasts (39). Related results were found in young and old rat MSCs. Here, increased cellular ROS production was observed with age. A hinting cause for the increased ROS level was the low superoxide dismutase (SOD) 1 (a central gene in the ROS response pathway) expression suggesting potential DNA damage (40). ROS is a known cause for DNA damage, from single base oxidation to single and DSBs, indicating that high ROS levels have an erroneous effect on genomic integrity (41).

### DSB Repair Efficiency, *In Vitro* and *In Vivo*

A similar approach was implemented on eyelid fibroblast cells originating from different ages of healthy donors, showing that the efficiency and quality of DNA repair through NHEJ and HR pathways decreased with age (42).

The role of faulty DNA repair machinery in age-related genomic instability was also found in *S. cerevisiae* and Drosophila. Mutations in the *sgs1* and *srs2* genes [encoding for RecQ helicase, homologous to the human *WRN* (43)] shortened *S. cerevisiae* life span through two distinct pathways: *sgs1*- and *srs2*-mutated cells stopped dividing randomly in an age-independent manner that required the RAD9 (cell cycle checkpoint control protein) DNA damage checkpoint, but late-generation *sgs1*- and *srs2*-mutated cells exhibited premature aging. The double sgs1/srs2-mutated yeast cells showed a high rate of terminal G2/M arrest. This arrest was suppressed by knockouts of *RAD51* (DNA repair protein RAD51 homolog 1), *RAD52* (DNA repair protein), and *RAD57* (DNA repair protein), hinting for malfunctioning HR. In a similar study, knockout of *DNA2*, encoding RecQ helicase-like protein, caused premature aging phenotypes including longer cell cycle time, transcriptional silencing, genomic alterations, and eventually shorter life span (44). Shaposhnikov et al. (45) used *D. melanogaster* to evaluate the effect of overexpression of DNA repair genes in several locations in the body and several time points during the life period on the Drosophila life span. Beneficial effects on life span were observed with overexpression of Hus1 (checkpoint clamp component), mnk (MAPK interacting protein kinases), mei-9 (meiotic 9, *D. melanogaster*), mus210 (Xeroderma pigmentosum, complementation group C, *D. melanogaster*), spn-B (spindle B, *D. melanogaster*), and WRNexo (WRN exonuclease, *D. melanogaster*), which control the processes of DNA damage recognition and repair (45). Myc, a key regulator protein of cell growth and proliferation, was shown to act as a pro-aging factor, probably by its ability to increase genomic instability. Overexpression of Myc in Drosophila increased the frequency of large genome rearrangements associated with faulty repair of DNA DSBs and decreased adult life span. *Myc* knockdowns demonstrated reduced mutation rate and extended life span (46). In aged mice, increased levels of DNA breaks or unrepaired DNA damage as illustrated by the formation of γH2AX (phosphorylated variant histone H2A) foci were observed (47–49). A positive effect on longevity was observed with overexpression of the human enzyme hMTH1 (MutT Human Homolog 1), which eliminates oxidized purine18 and deacetylase Sirt6 (50). Overexpression of SIRT6 promotes DSB repair by the activation of PARP1 [Poly (ADP-ribose) polymerase 1] and facilitating the recruitment of Rad51 (51) and NBS1 (Nijmegen Breakage Syndrome 1) (52) to DNA lesions.

### Evidence From Omics Experiments, *In Vitro*

The accumulation of genomic abnormalities is influenced by the quality of the repair pathways, which may also decline with age. Laurie et al. (53) studied age-related DNA damage in peripheral blood cells using single nucleotide polymorphism (SNP) microarray data from over 50,000 individuals. The frequency of detectable genomic abnormalities was low (<0.5%) at birth and rose to 2–3% in 50-year-old donors (53). Peripheral blood cells were also studied using whole-exome sequencing data from DNA of 17,182 individuals lacking hematologic phenotypes. Somatic mutations were rare in young donors (~40 years old) but became more frequent with age. Furthermore, while studying subjects at 70–79 years, compared with 90–108 years, mutation frequency rose from 9.5 to 18.4%, respectively (54). In some cases, the accumulation of damage was noticeable in relatively advanced ages and not as a linear progression. Goronzy et al. (55) found that memory T cells from healthy donors showed steady increase in levels of DNA damage in different ages, up to 65 years (55). All these findings lay the basis for longitudinal *in vivo* studies in model organisms to decipher the mechanistic view of this phenomenon (i.e., accumulation of DNA damage with age) in a manageable life span.

### DNA Repair in Long-Lived Animals

Analysis of two bat genomes showed that DNA repair and DNA damage signaling genes ATMh (human ataxia telangiectasia mutated), TP53 (tumor protein 53), RAD50 (DNA repair protein), and KU70 (XRCC6 protein product) are under selection in bats, suggesting that genome maintenance systems are under selective pressure in longer lived species (56). The study of Bowhead whales in the context of longevity is relatively new, but some insights have already been generated. Keane et al. (27) found duplications in genes linked to DNA damage repair and aging, such as *PCNA* (proliferating cell nuclear antigen). According to RNA-seq, both the PCNA copies were expressed. Several DNA damage and aging-associated genes, such as *ERCC1* and *ERCC3* (excision repair cross-complementing rodent repair), had unique mutations (compared to short-living animals) that were found to be under positive selection (27, 57). Mice with deleted *ERCC1* suffered from liver dysfunction and died prematurely before weaning, a phenotype that was rescued by overexpression of ERCC1 (58). It is interesting to notice that similar unique mutations in DNA repair genes (including *ERCC1* and *ERCC3*) were also found in naked mole rats and several species of bats (56, 59), hinting again at the role of DDR in longevity.

### Comparative Studies of Short-Lived and Long-Lived Animals

Long-lived organisms are suggested to possess more efficient genome maintenance mechanisms than short-lived ones. For instance, in a comparative study conducted on both short- and long-lived wild bats, the MMR system and the levels of DNA damage as well as the antioxidant enzymatic activities were compared (60). By analyzing the DNA MMR proteins MSH2 (DNA MMR protein) and MLH1 (MutL homolog 1) in the liver, lung, and brain of young, adult, and old bats, the study showed that the short-lived bats presented with a decrease in protein levels and an increase in microsatellite instability antioxidant activity with age while the long-lived bats exhibited higher levels of antioxidant enzyme activities. These results suggest that the antioxidant response of those animals is important to attain a long life span. Several genes associated with the repair of DNA damage have been reported as overexpressed in long-lived subterranean rodents than in short-lived surface-dwelling rodents. In addition, when comparing blind mole rats (the genus *spalax*) to rats, the long-lived *spalax* showed more transcript abundance in genes that encode for DNA damage repair proteins (61). In another comparative study performed on mice, naked mole rats, and humans, studying the expression levels of DNA repair genes in livers found that humans and naked mole rats exhibit higher levels of expression of DNA repair enzymes that are important for DNA damage sensing and the MMR, NHEJ, and the BER pathways (62). This evidence supports the hypothesis that long-lived organisms have better genome maintenance techniques than short-lived animals.

Antioxidants have been more attentively studied in naked mole rats than in bats. When comparing the activity of antioxidant enzymes such as SODs, catalase, and cGPx (human cellular glutathione peroxidase) in the livers of young, middle-aged, and old naked mole rats with mice, their activity was higher in at least one age class in mole rats (63). More importantly, Csiszar et al. found that relative expression of numerous antioxidant enzymes in naked mole rat blood vessels remained constant with age which may distinguish this species from other short-lived species, such as mice (64). Comparative *in vitro* studies were performed as well. One recent example of such a comparative study is the study performed by Ma et al. (59) which compared primary skin fibroblasts of 16 different mammalian species and highlighted differences in fibroblast profiles among long- and short-lived species (59). In contrast to these findings, the work of Page et al. (65) did not find correlation between DDR activity and life span. Page and Stuart (65) compared DNA repair rates and life span values by studying BER activity in brain and liver tissues from 15 species including mice, hamster, bat, sheep, dogs, pigs, and two bird species, quail and finch. The BER activity was found to be (negatively) correlated only with body mass (65).

### Contradicting Evidence, *In Vitro* and *In Vivo*

Despite the body of evidence mentioned here and in other reviews, some studies report contrary results. In a study performed by Schellenberg et al. (66), using long-term cultures of hMSC, Karyotype analyses at early passage and late passage did not reveal age-related chromosomal abnormalities and SNP array analysis did not reveal passage-related changes (66). A similar trend was observed when the efficiency of DNA MMR pathway was studied using CD4^+^ T cells from 25 to 80-year-old healthy donors. In this study, there was no connection between MMR frequency and donor’s age. Only when mutations were chemically induced, there was a negative correlation between MMR efficiency and age, but only among the younger age groups, 25–40 years old; no such connection was found for the older donors (67). Similar contradictions were also established in *in vivo* studies. Though there is a documented phenotype of DNA instability in aging yeast cells, it is still under debate whether accumulation of mutations is a cause of aging for yeast. Ijpma and Greider (68) found that chromosome loss was not related to loss of viability (68, 69). Daughter cells produced in early stages of their mother cell life live as long as their progenitors, yet cells produced later had reduced life span. However, the last cell created by a specific mother cell is still capable of bearing offspring. The observed increase in division time, which corresponded with an age-specific decline in reproduction in old mother cells, was only partially passed on to the daughter cells, and they resumed normal division time after a few budding cycles (70, 71). Kaya et al. (72) studied *de novo* mutations during multiple replications in daughter cells of mother cells at different ages. Mutations were found to increase with age, but their frequency was very low, and no effect on viability was detected (72). All these observations suggest genome integrity conservation through generations and question the role of genomic changes in aging in yeast. A possible explanation for aging-related genomic instability in yeast could be found in extra-chromosomal rDNA circles (ERCs), which were shown to be correlated with premature aging and short life span in yeast. *sgs1* mutant accumulated more ERCs than wild-type cells, causing shorter life span (73), while knockouts of FOB1 (DNA replication fork blocking protein) decreased the formation of ERCs and extending life span (74).

### Progeroid Diseases as Models for Aging

As mentioned earlier, age-related genomic instabilities in humans are studied through progeroid diseases. The first three genes causally linked to human aging *(according to HAGRID)* are progeroid phenotype causing genes: *LMNA* (Lamin A/C), *WRN* (Werner Syndrome RecQ-Like Helicase), and *ERCC8* (DNA excision repair protein) (75). *LMNA* is a gene coding for a nuclear envelope scaffolding protein, mutations in which lead to genomic instability which in turn cause HGPS. This syndrome serves as a model for human aging since progerin (the mutated LMNA protein) can be found in normally aging cells and is believed to cause cellular toxicity and senescence (76). Mutates *WRN* (RecQ-like helicase) causes Werner syndrome and is involved in the DNA DSB repair pathway, similar to the *S. cerevisiae SGS1* (43, 77). *ERCC8*, mutated in Cockayne syndrome patients, is a protein involved in the NER pathway, mutations in which cause high sensitivity to UV due to loss of ability to repair UV-induced DNA damage (78). These genes exemplify the effect of the DNA damage repair quality on aging, as brought forth by the previously mentioned *in vitro* and *in vivo* studies. Besides these three genes, another, more recently described gene is the *SPRTN* (SprT-Like N-Terminal Domain) gene whose translated protein product acts in the translation repair pathway, allowing DNA replication despite single nucleotide lesions. Mutations in this genes cause Werner-like progeria, probably due to their disabling effect on this replication pathway (79). Additional support for the importance of genomic integrity in the aging process is 53BP1 (p53 binding protein 1) (76). This protein is crucial for DNA DSB repair mediation and proteins’ recruitment. First described as a p53 binding protein, 53BP1 recognizes DSB histone code and recruits the repair proteins to the site in different mechanisms depending on different stages of the cell cycle (80). The DNA DSB repair is crucial as it is well established that DSBs lead to premature aging and senescence (81, 82).

## Telomere Alterations and Cellular Senescence

Besides direct DNA damage, cellular senescence can be induced by diverse mechanisms, the principal among them is telomere attrition. Telomeres are short tandem repeats that serve as “caps” that protect the ends of the chromosomes from being recognized as DSBs and prevent the cascade of DDR in the cell and actively participate in genome maintenance. With every cellular division, the telomeres shorten by several repeats.

### Evidence From *In Vitro* Studies

In most organisms, telomere elongation is controlled by the enzyme telomerase under tight regulation to ensure sufficient number of replications, yet when this number is reached, telomere elongation is seized (2, 83). Once telomeres reach the critical length, the cells undergo senescence and stop proliferating (84). This process is believed to be the trigger for the aging process, according to the telomere theory (11, 85, 86). It is further supported by Bodnar et al. who proved that telomere elongation caused by ectopic expression of telomerase avoids the senescence phenotype (87). His work relied on one of the earliest studies linking telomere shortening to aging which was performed by Harley et al. on human fibroblast cells (88). In their paper, they describe the shortening of telomeres in aging fibroblasts alongside chromosomal abnormalities, specifically the fusion of two chromosomes at the telomeric region and chromosomal rearrangement, while hinting at a biological significance to the shortening process. Since this early study, numerous studies have emerged strengthening this association and aiming to elucidate the exact underlying mechanism of telomere shortening. Murillo-Ortiz et al. (89) studied telomere alterations using T, B, and NK cells from 20 to 25-year-old and 60 to 65-year-old donors. Treatment with concanavalin A (a mitogen of T cells) caused increase in telomere length and number of replications in the samples from the young donors, but did not improve the samples from the older donors, which exhibited loss of telomere parts, decrease in telomere length, and decreased proliferation potential (89). Age-related changes in telomere length were also established in bone marrow hMSC in a long-term *in vitro* study (90). *COMET* assay revealed higher levels of damage in cells from older donors (91). Similar results were obtained in the study of CD34^−^ and CD34^+^ cells isolated from healthy donors of different ages. However, some of the cells exhibited telomere shortening that was not correlated with age. It seems that CD34^+^ cells from older donor suffer from increased non-telomeric DNA damage, but the variation among the cultures hints for multiple factors contributing to DNA damage (92).

### The Question of Telomere-Related Senescence in *S. cerevisiae*

For *S. cerevisiae*, various studies were performed on the effect of missing/broken telomere and mutated telomerase on the physiology of the organism. Genetic manipulations of *S. cerevisiae* cells caused decreased growth, irregular shape, and eventually, cellular senescence (69). Several genes, such as *EST1* (telomere elongation protein), *EST2* (telomere reverse transcriptase), *EST3* (telomere replication protein), *TLC1* (template RNA component), *RAD9, RAP1* (DNA binding protein), *CDC13* (cell division control protein 13), *TEL1* (serine/threonine protein kinase), *MEC1* (serine/threonine protein kinase), and *MRC1* (macrophage mannose receptor 1 precursor) were studied in connection to telomere-related senescence; however, despite the extensive experimental work put into using mutated cells, the role of eroded telomeres in “natural” cellular senescence in yeast remained questionable (93). For example, *EST1-4* (ever short telomere) mutants began to lose viability after 60 doublings, but late knockout cultures continued to maintain proliferation potential (94). Cells with mutated telomerase exhibited irregular morphology and short telomeres, but these changes did not cause deadly damage and determinate senescence (95). One hypothesis connects aging to telomere erosion through the transcription of subtelomeric genes. Genes located in subtelomeric regions are affected by transcriptional silencing which was found to change in an age-related manner. Kim et al. (96) found that silencing of genes in subtelomeric regions declined during the cell’s senescence, hinting at a connection between the transcription of subtelomeric regions and cellular senescence in yeast (96). The work of Austriaco and Guarente (97) reinforced this model, as they found that mutated telomerase extended life span (relatively to the wild type), probably by hanging the silencing procedure in the subtelomeric locations (97).

### Telomere Alterations in *C. elegans*

The evidence for the role of telomere attrition in the senescence of *C. elegans* are contradicting and are influenced by the worm’s unique physiology, as the adult worm go through a short reproductive stage, followed by a “post-mitotic life” with a definite number of steady post-mitotic cells (98, 99). Overexpression of HRP1 (Heterogeneous nuclear Ribonucleo Protein 1) was found to increase telomere length and, subsequently, the life span of transgenic worms. The resulting prolonged life span was reliant on *DAF16* (Forkhead box protein O gene, *C. elegans*) (100), which codes for a FOXO (Forkhead Box protein O) transcription factor and is required also for the effect of the insulin/IGF-1 pathway on life span in *C. elegans* (98). This connects to the first life span-related gene that was discovered in *C. elegans*—*AGE-1*. *AGE-1* encodes a phosphatidylinositol-3-kinase that functions in the insulin/IGF-1 signaling pathway. Mutations in this gene cause delay in age-related deterioration of body movement and muscle deterioration a twofold extension of the life span (17, 101, 102). Opposing results were obtained by Raices et al. (103) that found no correlation between telomere length and the life span of *daf-2* and *daf-16* mutants. Furthermore, a study of different wild-type populations with diverse telomere lengths found again that the length of the telomeres was not correlated with life span (103). Similar phenomena were observed in mutants of *TRT*-1, a catalytic subunit of telomerase. The mutants reproduced regularly for several generations but eventually became sterile (104). The telomeres shortened by ~125 nucleotides per generation and suffered from sequence abnormalities, but the mutation and other telomere-shortening manipulations did not affect post-mitotic aging (104, 105). Mutations in *MRT-2*, a gene in the same pathway as *TRT-1*, caused similar phenotypes including telomere shortening, accumulation of DNA damage, and sterility. Similarly, the mutation had no effect on life span (106).

### Relevance of Drosophila and Mice in the Study of Telomere-Related Senescence

While most organisms have a tandem repeat-based telomere and a telomerase for its maintenance, Drosophila telomeres are composed of randomly ordered retrotransposable elements that are maintained by retrotransposition (107–110). Although the length of the drosophila telomere is close to the human telomere (~10–12 kb), its structure is much more complex since each building block contains its own promoter regions, coding sequences, and regulatory elements (110, 111). These might be the reasons why there are no evidence for connection between telomere shortening and aging in Drosophila. Walter et al. (112) found that like *C. elegans*, the length of the telomeres in Drosophila did not affect life span, but it was correlated with fertility and fecundity (112). Study of age-related transcriptional changes did not find any telomere-related modifications (113). As in *C. elegans*, the FOXO-mediated insulin/IGF-1 pathway can affect the Drosophila life span (114), but a possible connection to telomere length was not studied.

Similar to Drosophila, the relevance of mice telomeres studies is also debated and unclear. Several studies show that mice with shortened or lengthened telomeres exhibit decreased or increased life span, respectively (115–119). The premature aging of telomerase-deficient mice was reverted when telomerase was genetically reactivated in aged mice (120), and systematic viral transduction of telomerase in adult wild-type mice delayed normal physiological aging (121). Mice with telomerase deficiencies exhibited signs of accelerated aging, but only after several generations and that overexpressing telomerase did not alter aging (122). The delayed phenotype implies that for senescence activation, telomeres need to be shortened extensively, in a manner that might not be realistic during the regular mouse life span. Mice are interesting models for the research of human telomere diseases. Telomerase dysfunction in humans causes a disease called dyskeratosis congenita (DKC), which shares some features with telomerase-deficient mice (123). However, the use of mice as a model for telomere-related human aging and aging-related human diseases is very questionable since the telomeres of most laboratory mice are 5–10 times longer than in humans (~40–50 kb), yet their life span is 30 times shorter (111, 124). Like *S. cerevisiae*, although genetic manipulations of telomere and telomerase may influence the organism’s life span, this effect might be overlooked while observing naive mice.

### Telomere-Related Senescence in Long-Lived Animals

In a study conducted on four wild populations of long-lived bats, telomeres were shown to maintain their length in blood fibroblasts in the *M. myotis* species, and similar to humans, they also showed no signs of telomerase expression (125). In naked mole rats, genes involved in the function and regulation of telomerase, Tep1 (telomerase-associated protein 1) and Terf1 (telomeric repeat binding factor 1), were found to have undergone positive selection which may contribute to their slow rate of aging, though contradicting results were also published (126). For instance, a different study established that similar to mice (but unlike humans), naked mole rat somatic cells express telomerase, although at lower levels, and are not amenable to telomere-dependent replicative senescence. Gomes et al. (124) studied the telomeres of the bowhead whale lung fibroblast cells and found that the average telomere lengths was ~9 kb, in resemblance to human telomere length (124). The bowhead whale telomerase had repressed activity as well, again, similar to human telomerase (124, 127). Lai et al. (128) tested cultured bowhead whale lung fibroblasts at different population doublings and found age-related telomere shortening (128).

### Human Diseases—Telomeropathies

In humans, early telomere attrition or exhaustion leads to telomeropathies (telomere syndromes) and age-related diseases (129). Telomeropathies are divided into two subgroups: primary and secondary telomeropathies. Primary telomeropathies are disorders of impaired telomere maintenance, or in other words, telomere disorders, while secondary telomeropathies are disorders in which the main mutated gene has a role in DNA repair, thus affecting telomere maintenance without actual damage to the telomere maintenance biology (130, 131). As previously mentioned, human genetic diseases are the main mode of “*in vivo*” research in humans. Almost all secondary telomeropathies, such as Werner syndrome and Hutchinson–Gilford progeria, are associated with premature aging and increased disease risk. Yet, most of the primary telomeropathies, such as the various forms of DKC, do not present with a progeroid phenotype but do have a wide phenotypic range which includes bone marrow failure, hair loss, emphysema, liver cirrhosis, osteoporosis, and pulmonary fibrosis. All these symptoms are also associated with aging, linking once again, the deterioration of bodily functions to shortening telomeres (130). A study conducted on 274 pairs of aged twins concluded that shortened telomeres can forecast death in the elderly (132). There are supporting (133, 134) and contradicting (135–137) evidence for this, yet the authors used intrapair comparisons on same-sex twins in order to eliminate biases of gender, genetic background, and age differences, providing another strong supportive evidence.

### Telomere Position Effect—Over Long Distances

An additional effect of telomere shortening is the increase in expression of TPE-OLD (Telomere Position Effect—Over Long Distances) genes. Robin et al. demonstrated, using high-resolution Hi-C (an unbiased 3D chromatin capture technique), that long telomeres form chromatin loops reaching up to 10 Mb away from them. This loop is highly condensed causing epigenetic silencing of the genes in that region (called TPE-OLD genes). When the telomeres shorten, this loop is no longer able to form and in turn, the epigenetic regulation is changed to activation of the TPE-OLD genes. This happens before the telomeres reach the critical length that causes activation of DDR, thus leading to another earlier possible effect of telomere shortening on aging (138, 139). Interestingly, a following study by Kim et al. showed that one of the TPE-OLD sensitive genes is *hTERT*, the core reverse transcriptase component of telomerase (140). This is also supported by the abovementioned studies of subtelomeric regions performed in yeast.

## Senescence-Related Epigenetic Alterations

Epigenetics as a field, and specifically epigenetics of aging, has gained much interest in recent years. According to Pal and Tyler (141), genetics only explain 20–30% of the aging phenomenon and researchers now aim to elucidate the remaining 70–80% mainly through epigenetics. Epigenetics can be broadly defined as changes in gene regulation without changes to the DNA coding sequence. It encompasses a range of possible changes; DNA methylation (142), histone modifications (143), various non-coding RNAs (144), and recently emerging evidence show that change in chromatin structure offers epigenetic regulation as well (145).

### DNA Methylation

Age-related epigenetic modifications were shown in long-term cultures of hMSC. DNA methylation profiles of early and later passage were compared and revealed highly consistent senescence-associated (SA) modifications at specific CpG sites (66). Similar results were obtained in a long-term *in vitro* study of bone marrow hMSC. DNA methylation analysis revealed methylation changes between early and advanced passages. At early passages, 61.6% of all CpG islands were methylated while later, methylation decreased to 44.7% (90). A related phenotype was also observed in skeletal muscle stem cells (SCs) from young and old mice. Epigenetic profiles revealed age-related accumulation of epigenetic changes (145). Additionally, DNA methylation profiles were compared between different passages in order to identify SA changes. 1,702 CpG sites were SA hypermethylated, and 2,116 CpG sites were SA hypomethylated. SA hypermethylation was enriched in inter- and intragenic regions, and in the 3′UTR, while SA hypomethylation was highly enriched in intergenic regions (146).

The gene *dDNMT2* (DNA methyltransferase) was found to be necessary for maintenance of the average life span of the flies, as mutants suffered from shorten life span. Overexpression of *dDNMT2*, however, extended *Drosophila* life span (147).

DNA methylation is also used as an “aging clock” to predict a person’s age. Horvath has provided a breakthrough “epigenetic clock” in his study from 2013. He used 8,000 samples from 82 publicly available datasets of Illumina DNA methylation arrays, including 51 tissues and cell types. This clock was able to detect the age of the sample using only 353 CpGs (148). This remarkable clock was later further improved, using fresh human blood samples, and now contains just three CpG sites (149).

### Age- and Radiation-Related DNA Methylation, *In Vitro*

Koch et al. (146) studied age-related methylation profile in bone marrow hMSCs under several conditions and after different number of passages. Their results reveal that ionizing radiation (IR), although connected to DNA damage, did not affect age-related methylation profile. Chemical immortalization of the cells increased telomere length, but the cells still exhibited a senescence-related methylation profile. The only treatment that completely inhibited the age-related profile was “reprogramming” the cells back to their pluripotent stage (induced pluripotent stem cells) (146). It seems that although senescence has an epigenetic regulation, IR and immortalization are not connected to this process.

### Histone Deacetylation—Sirtuin 2 (SIR2) and RPD3, *In Vivo*

Epigenetic alterations were also found to play a major role in *S. Cerevisiae, C. elegans*, and *Drosophila* life span. The histone deacetylase SIR2 was found to extend yeast life span when overexpressed, as was found in worms and flies (150, 151). A double mutant of the *C. elegans SIR2* homolog significantly induced life span, and analysis revealed that the *sir-2.1* functions upstream of *daf-16* in the insulin-like signaling pathway (152). Also, it was found that during aging, histone H4K16 acetylation increases while H3K56 acetylation decreases (153). This is thought to be a result of the decline of SIR2 that occurs naturally during aging, which leads to H4K16 deacetylation (154). Moreover, all histone protein levels were found to descend with age which has a direct effect on the life span of the cells (155). RPD3, another histone deacetylase targeting H4K16, was also found to affect longevity in several organisms. *RPD3* deletion increased *S. cerevisiae* life span by increasing silencing at three loci, the silent mating type *(HMR*), subtelomeric, and rDNA loci (96). Similarly, a fractional decrease in the levels of Rpd3 resulted in a 30–50% increase in life span of Drosophila (156, 157). Yet, Drosophila life span was not affected through gene silencing. It seems that in flies, the two deacetylases, SIR2 and RPD3, function opposingly at the euchromatin influencing gene expression and affecting longevity (156).

### Age-Related Histone Deacetylation—Sirtuin Family, *In Vivo*

In mice, numerous sirtuin paralogs were found to improve different characteristics of aging (158, 159). Transgenic overexpression of SIRT1, an ortholog of the histone deacetylase SIR2 in yeast, improved healthy aging but did not increase longevity (160). The mechanisms involved in the beneficial effects of SIRT1 are complex and interconnected, including improved genomic stability (161, 162). Other convincing evidence for the sirtuin role in pro-longevity is the SIRT6 that modulates genomic stability through histone H3K9 deacetylation (163–165). Mutant mice that lack SIRT6 exhibit accelerated aging (166), while overexpression in male transgenic mice leads to longer life span compared to wild-type animals, an effect that is associated with reduced serum IGF-1 (Insulin Growth Factor 1) and other indicators of IGF-1 signaling (50). It has been reported that SIRT3 improves the regenerative ability of aged hematopoietic stem cells (167). Therefore, in mice, SIRT1, SIRT3, and SIRT6 contribute to healthy aging. SIRT6 has been associated with aging and disease protection through repression of aging and cancer-related transcription factors, promotion of chromatin changes essential for DNA repair, maintenance of telomere structure, and thus preventing genomic instability and senescence, in humans as well (168).

### Histone Methylation, *In Vivo*

Greer et al. (169) discovered a crucial role for histone methylation in aging. They examined chromatin in different states and its effect on life span by investigating different enzymatic complexes and performing a targeted RNAi screening in fertile *C. elegans*. They discovered what is now known as the COMPASS complex, a key regulator of worm life span that acts in germline cells. This complex trimethylates histone H3 at a lysine residue (H3K4me3), and deficiencies in its members including the H3K4 methyltransferase SET2 extend life span (169). On the other hand, loss of function of the H3K4 demethylase RBR2 leads to a decreased life span, which agrees with the key idea that an increase in H3K trimethylation activates chromatin, thus promoting aging. When studying histone marks associated with repressed chromatin, Maures et al. discovered that absence of the demethylase for the repressive H3K27me3 mark—UTX1, increased worm life span separately of the germline. This mark significantly declines with normal aging in soma cell, which means that repressive H3K27me3 levels allow somatic maintenance during aging (170). Related phenotypes for H3K4me3 were also discovered in Drosophila. Overexpression of *LID*, a RBR*2* homolog, extends life span, while its knockdown shortens life span of male flies by 18% (171). Siebold et al. (172) found that heterozygous mutations in two core subunits of PRC2 (Polycomb Repressive Complex 2), the histone H3 lysine 27 (H3K27)-specific methyltransferase E(Z), and the H3 binding protein ESC, enhanced life span and decreased H3K27me3 levels in adults. Mutations in *trithorax* (*trx*), an antagonist of Polycomb silencing, reversed the H3K27me3 level of the *E(z)* mutants and suppressed their enhanced longevity and resistance to oxidative stress and starvation, hinting that the reduced levels of H3K27me3 are connected to longevity and stress resistance in the PRC2 mutants (172). In drosophila, H3K27me3 seems to influence life span in an opposite manner compared to *C. elegans*. Mutations in H3K27 methyltransferase (PRC2) subunits E(Z) and ESC reduce global levels of H3K27me3 and extend life span of male drosophila by activating target genes *Abd-B* (abdominal B) and *Odc1* (Ornithine Decarboxylase 1) (172).

### Large-Scale Chromatin Remodeling, *In Vitro*

Epigenetic alterations include also genomic organization and large-scale chromatin remodeling which are facilitated by smaller scale epigenetic changes such as DNA methylation and histone post-translational modifications (PTMs). Human MSCs were also used in a recent study performed by Dillinger et al. (173) showing genomic organizational changes associated with senescence. In this study, they show using Hi-C data that there is little change in nucleolus-associated chromosomal domains between proliferating and senescent cells, yet there are large satellite repeat clusters that dissociate from centromeric and pericentromeric regions in the nucleolus during senescence (173). These findings relate back to the established aging-associated genomic instability and chromatin remodeling as discussed earlier.

### Chromosomal Rearrangements, *In Vivo*

An examination of chromatin structure during aging in Drosophila revealed significant age-associated chromosomal rearrangements (174). In young flies, H3K9me3 and HP1 were enriched in the pericentric regions, in chromosome 4, and in heterochromatin islands spread throughout the genome. However, this enrichment decreased in an age-associated manner, equalizing H3K9me3 and HP1 levels in the pericentric regions, chromosome 4, heterochromatin, and euchromatin. Furthermore, single-cell immunohistochemistry showed changes in nuclear distribution of H3K9me3 and HP1 marks with age.

### miR’s Activity, *In Vivo*

miR’s also play a role in aging. Liu et al. (175) showed that miR-34 regulates age-related effects and long-term brain stability in *Drosophila*. Expression of the drosophila *mir-34* exhibits adult-onset, brain-enriched, and age-related phenotypes. While *mir-34* loss induced genetic profile of brain aging, late-onset brain degeneration, and a significant decline in life span, *mir-34* upregulation extended life span and reduced neurodegeneration evoked by human pathogenic polyglutamine disease protein (175). miRNAs also affect gene expression during the aging process in mice (176) and modulate senescence in human cell lines (177). Studies have found that miRNAs work in groups by modulating gene expression and silencing that can lead to age-dependent disease states or alternatively to longevity (178). Inherited epigenetic effects in miRNA loci cause changes in gene expression that modulate longevity (179), and miRNAs that target the insulin/IGF-1 pathway can foresee up to 47% of life span variations (180). Some loci show positive effects on life span, promoting longevity, while others show the opposite effect, causing a shorter life span (181). Ugalde et al. have reported that alteration in the expression of two miRNAs leads to a progeroid phenotype in a mouse model for a progeria syndrome by effecting key components of the DNA-damage response pathways (182).

### Epigenetic Alterations in Long-Lived Animal Model

Only a few studies were conducted on the epigenome of the naked mole rats, especially in the context of aging. Sequencing the naked mole rat genome (183) showed that its genome had relatively low CpG density and higher fraction of CpG dinucleotides within CpG islands compared to the human genome. CpG dinucleotides within CpG islands contribute less to genetic variation because of their lower methylation rate. In a different study of the reprogramming of naked mole rat cells, analyzing the global histone landscape revealed that naked mole rats had higher levels of repressive H3K27 methylation marks and lower levels of activating H3K27 acetylation marks than mice which suggests that naked mole rats display a more stable epigenome that resists de-differentiation contributing to its longevity as well as to its resistant to cancer.

### DNA Methylation—Twin Studies

Since the genomic methylation profile of each person is unique, comparative studies are needed. Monozygotic (MZ) twins have identical methylation and epigenetic patterns immediately after birth and in early childhood, making them a perfect platform for the study of methylation and epigenetic changes in general. Such a study performed in 2005 by Fraga et al. has provided many insights on the genomic methylation and gene expression changes in MZ twins of different ages. Fraga et al. were the first to look into epigenetics of MZ twins, and in their paper, they described the changes in methylation with age between the twins as “epigenetic drift.” Epigenetic drift, as they define it, is changes in the methylation profile over time due to accumulating “small defects” in transmitting epigenetic information over successive cell divisions. In other words, changes in the epigenome of an organism over time are due to random changes in methylation (184). The effect of epigenetic drift on the genome can be small or large, depending on where those changes occur. Keeping in mind that hypermethylation of promoter regions is associated with transcriptional repression, epigenetic drift can, and indeed does, cause changes in gene expression. The pattern of elevated methylation with age was also shown for general human populations (not twins) by Horvath (148) and Hannum et al. (185).

### Histone PTMs

Human studies of histone PTMs related to aging are emerging and following are a few recent advances. There is accumulating evidence to the role of histones in memory and cognitive functions (186, 187) in the human brain. Hohl et al. showed that the histone methyltransferase SUV39H1 plays a role together with HDAC4 (histone deacetylase 4) in repression of pro-hypertrophic genes in the human heart (188) linking histone PTMs to cardiac stress and aging. Ucar et al. most recently published results indicating association of chromatin condensation with age in 27 histone-related genes. Among those genes were a few coding for histones (HIST1H3D, HIST1H3E, and HIST4H4) and histone modifiers such as EZH1 and SETD7 (189). These results strengthen the previously established patterns of reduction in core histone expression and changes in histone modifications (190).

## Conclusion

Healthy aging and cellular senescence are complex processes of great interest to researchers. The multigenic nature of both of them complicates studies and necessitates creative and novel approaches in the path for understanding those phenomena. The three spear-headed strategies implemented for this purpose have brought forth much information and knowledge, yet there is still much to learn in these fields. The doubting and contradicting results in *in vivo* studies are influenced both by physiological and genetic differences between the model organisms and humans and the differences in the possible research methodologies between *in vitro* and *in vivo* studies. In many cases, the age-related phenotypes searched for and studied *in vitro* are not visible *in vivo* or not relevant for the model organism (Table 1.).

**Table 1:** Evidence for correlation between DNA damage accumulation, telomeres attrition and epigenetic alterations, and aging in *In Vitro, In Vivo*, and aging-like human syndromes studies.

	Age-related accumulation of DNA damage	Telomere attrition	Epigenetic modifications
Cell cultures (human and mice)	+	+	+
*Saccharomyces cerevisiae*	Debatable (contradicting results)	−	+
*Caenorhabditis elegans*	−	Debatable (contradicting results)	+
*Drosophila melanogaster*	+	−	+
*Mus musculus*	+	−	+
Human	+	+	+
*Heterocephalus glaber* (NMR)	+	Contradicting results	N/A
Bats (spp. *Myotis*)	+	+	N/A
*Balaena mysticetus*	+	+	N/A

Molecular processes such as DNA damage repair, telomere shortening, and epigenetic alterations discussed earlier are the driving forces of the aging process in human, but their significance is varied in other organisms. Many evidence for age-related accumulation of DNA damage were found in *in vitro* studies, both in human and mice cell cultures. The connection between DNA damage and aging is emphasized by the secretion of senescence-associated proteins during cellular senescence, a phenotype which is activated by DNA damage and is common for both human and mice. Human progeroid diseases also show the connection between early aging and faulty DNA repair. In yeast, flies and mice, however, although some evidence for age-related damage and faulty DNA repair mechanisms were found, contradicting and debating results highlight the complexity of the use of these model organisms in this aging research. The study of telomeres in relation to aging demonstrates the questions derived from both physiological differences between organisms and differences in research approaches. The connection between telomere attrition and aging is very present in human aging (both in *in vitro* studies and as telomeropathies such as DKC, Werner syndrome, and Hutchinson–Gilford progeria) but not relevant in model organisms. In *C. elegans*, the evidence are contradicting. In drosophila, maybe because of the unique telomere structure, there are no evidence connecting telomere attrition to aging. In yeast and mice, genetic manipulations enabled the study of telomere-aging relations, but such relations were not seen in wild-type subjects. The study of telomere-related aging in mice especially feature the difficulties of comparing human and model organisms, since the telomeres of most laboratory mice are 5–10 times longer than in humans, but their life span is much shorter.

Interestingly, the only common effector on aging found among cell cultures, different model organisms, and humans is epigenetic modifications. Epigenetic modifications are indeed a part of every genetic response in the cell, but the existence of common age-related modifications and key-players is intriguing. Epigenetic alterations are “core” elements in cellular responses. They play an upstream role to specific cellular processes, and this might be the reason for the relatively joint phenotypes. Furthermore, epigenetic modifications that are related to age-associated chromosomal rearrangements in yeast and flies might be a link to age-related DNA damage, where direct evidence were not found.

Though much progress has been achieved, full understanding of these mechanisms has still a long way to go. New tools such as GWAS and EWAS studies hold the potential to further elucidate the aging phenotype by investigating large datasets obtained from human subjects, but, it is still important and useful to study the above discussed strategies and organisms. However, the selection of those organisms will have to be more conscious and target-based.

## Author Contributions

GL, DG, and HS wrote and edited the article; LS and GA edited the article.

## Conflict of Interest Statement

The authors declare that the research was conducted in the absence of any commercial or financial relationships that could be construed as a potential conflict of interest. The reviewer AP and the handling Editor declared their shared affiliation.
